# Designing reproducible large-language-model-assisted scientific analyses

**DOI:** 10.1016/j.patter.2026.101644

**Published:** 2026-09-04

**Authors:** Casey W. Dunn, Darrin T. Schultz, Jacob M. Musser

**Affiliations:** 1 Department of Ecology and Evolutionary Biology and Quantitative Biology Institute, Yale University, New Haven, CT, USA; 2 Department of Biological Sciences, Lehigh University, Bethlehem, PA, USA; 3 Department of Molecular, Cellular, and Developmental Biology, Yale University, New Haven, CT, USA

**Keywords:** reproducibility, large language models, scientific workflows, provenance, determinism, research software, data analysis, AI in science, open-weight models, reporting standards

## Abstract

Large language models (LLMs) increasingly write code, analyze data, and orchestrate scientific workflows. This can create reproducibility challenges because LLMs blur the boundary between how an analysis is built and what it depends on at run time. Guidelines for LLM-assisted science begin with a critical choice: whether the LLM sits *on* the data path of the published analysis, a live step results depend on, or *off* the data path, producing durable artifacts such as code. Reproducible analyses require preserving data, code, and runtime; an on-path LLM becomes part of the runtime, a dependency that may change, be deprecated, or become inaccessible. We derive six recommendations: (1) keep the LLM off the data path where possible, (2) preserve LLM-generated artifacts, (3) verify results by methods suited to the LLM’s role, (4) consider open-weight models, (5) record the model version, and (6) assess determinism.

## The bigger picture

Scientists are rapidly adopting large language models (LLMs) to write code, process data, and even draw conclusions. This raises multiple questions about how to enhance the reproducibility of AI-facilitated research. Decades of work on reproducibility have shown that reproducing a computational result requires preserving its data, code, and runtime. An LLM invoked as part of an analysis becomes part of that runtime, yet many of today’s frontier models are closed and short lived, so an analysis that depends on one may not be reproducible even a year later. This tutorial offers an organizing question—whether the LLM sits *on* or *off* the data path of the published analysis—and explains how the familiar goals of reproducibility, provenance, determinism, and interpretability carry over to LLM-assisted work. The aim is to help researchers deliberately reason about AI use with an eye toward the best practices in data analysis that have always mattered.

## Teaser

Researchers are rapidly adopting large language models (LLMs) in their work, but this can create challenges for scientific reproducibility. In this tutorial, the authors offer an organizing question—does the LLM sit on or off the data path of the published analysis? They then explain how this question, combined with familiar goals of reproducibility, provenance, determinism, and interpretability, can help researchers deliberately reason about their AI use and preserve best practices.

## Introduction

Large language models (LLMs) are quickly becoming a powerful and widespread tool in scientific data analysis. Early LLM applications were primarily conversational (“chatbot”) systems that generated text in response to user prompts. More recently, LLMs have been extended through harness frameworks that connect them to memory, external tools, and iterative workflows. These systems, often referred to as *agents*, allow LLMs to carry out multi-step tasks such as literature searches, code writing, data analysis, and hypothesis generation while maintaining information across interactions. Table 1 collects the terminology this paper uses.

**Table 1:** Terminology used throughout this paper

Term	Meaning
**Reproducibility properties**	

Reproducibility	another investigator, with reasonable effort, can obtain the same results by rerunning the same code on the same data
Determinism	repeating the same step with the same inputs produces the same output
Provenance	a structured record of how a result was produced: the inputs, operations, and conditions involved
Interpretability	the ability to inspect and understand how a method arrived at its output

**The LLM and its parameters**	

LLM (large language model)	model trained on large amounts of text that produces text in response to text
Weights	numerical parameters a model learned during training—tens to hundreds of billions of numbers
Open-weight/closed-weight	whether those weights are publicly available (open) or kept private by the vendor (closed)
Inference stack	the hardware and software layers that actually run a model to produce output
Sampling parameters	settings that control how randomly the model chooses each next token; common ones are *temperature*, *top-p*, and a random *seed*
Context	the full text the model reads on a single call—prompts, prior exchanges, loaded skills, attached data
Context window	the maximum amount of context a model can read at once, measured in tokens (roughly, word fragments)

**LLM use**	

Application programming interface (API) call	a programmatic request to an LLM, typically sent by software or a harness, that returns model output; API calls enable automated, repeated, or batched interactions and are commonly used when an LLM operates on the data path
Prompt	the instructions and accompanying text given to an LLM on a single call
Skill	a reusable, file-based package of prompt instructions an LLM can load on demand
Harness	software around an LLM that assembles prompts, maintains memory across calls, invokes tools, and sends the requests to the model
Agent	a harness plus an LLM, configured to carry out a task with some autonomy
Tool call	a call to an external function that the LLM requests—running a script, querying a database, and reading a file

**Software-engineering terms**	

Runtime environment	the software environment (interpreters, libraries, and operating-system facilities) that executes code when you run it
Container	a self-contained bundle of code plus everything it needs to run, built with a tool such as Docker or Apptainer, so it behaves the same on any computer
Version control	tracking changes to files with a tool like git, so any earlier state can be recovered
Bit-for-bit determinism	output identical down to the last digit across runs

LLM-based scientific agents are being developed for a wide variety of tasks, ranging from experimental design to data interpretation.^1^ An LLM-based agent may synthesize prior findings from the literature, identify relevant datasets from public repositories, and write software to analyze them. It may then execute and monitor the analysis, troubleshoot failures, and invoke specialized tools for subtasks such as image classification, object detection, or data standardization. Agents can also coordinate teams of subagents in parallel and integrate the results.

**Figure 1:**
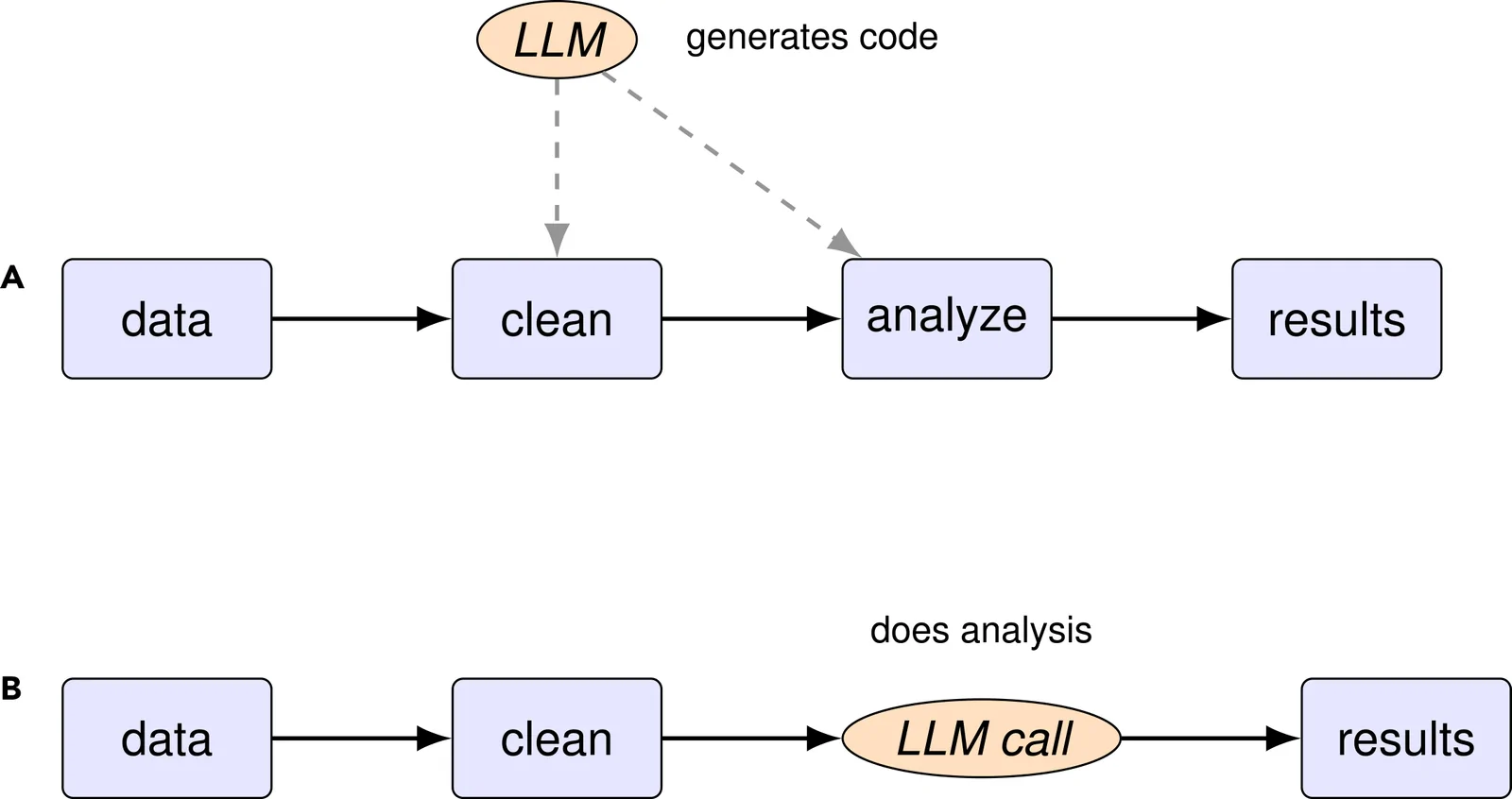
Example analysis-workflow graphs (A) The LLM lives off the data path: the assistant produces code that can be versioned and archived, and the code itself sits on the path. (B) The LLM lives on the data path: the model call is itself a node, and reproducing the workflow requires the original runtime.

Where the LLM sits in the analysis has major implications for reproducibility. An analysis workflow can be thought of as a graph (Figure 1): each step is a node, and the edges represent the flow of data between steps. The path from data inputs to output results is the *data path*. An LLM is *on* the data path when data move through it at run time, for example when the LLM performs image segmentation or classification. An LLM is *off* the data path when it is used to produce an artifact that sits on the path, for example when it writes code that is then committed to the pipeline. A useful operational test is whether the published analysis can be rerun from inputs to results without invoking any LLM: if yes, the LLM is off the data path; if no, it is on it. Nearly everything that follows turns on this distinction.

The central question of this paper is how LLM components in scientific data analysis should be designed and shared to maximize reproducibility. Code written in a programming language can, in principle, be archived in a form that allows it to be re-executed years later with the same results. Decades of work on computational reproducibility have been directed toward this goal.^2^^,^^3^^,^^4^ LLM components, especially those backed by frontier closed-weight models with vendor-controlled lifetimes, do not yet offer the same guarantees.

The need to improve reproducibility of workflows that include LLMs mirrors a familiar pattern in science: as informal, difficult-to-reproduce approaches mature, shared standards and practices arise to codify them (such as git-based management for analysis code and requirements to deposit data in specific public archives). For LLMs and LLM-based agents, the same need is now present. For example, frequently used instructions and supporting resources can be packaged as version-controlled *skills*, making them easier to inspect, reuse, and archive.

Our aim here is to formalize the challenges of reproducible work with LLMs within the framework established for traditional software, and to make specific recommendations. Our intended audience is scientists who have begun deploying LLMs in their research and are familiar with the general basics of their use, but who would like to step back and ensure that their use is scientifically robust. We do not argue for or against LLM use in data analysis. We do suggest that any LLM component of an analysis, whether a reusable prompt package, a bespoke prompt, or a direct application programming interface (API) call, should be designed with the same formal care as any other component. In this sense, where reproducibility is concerned, we should treat LLMs as a normal technology.^5^

## Reproducibility requires data, code, and runtime

An analysis is reproducible when another investigator, with reasonable effort, can obtain the same results the authors report. We use *reproducibility* in this narrow sense throughout (rerunning the same code on the same data), distinct from *replicability* (the same finding under new data) and *robustness* (the same finding under different methods). This is old ground in computational science, and a considerable literature has accumulated on what it takes in practice.^2^^,^^3^^,^^6^ The rough consensus reduces to three ingredients. One needs the *data*, in a form that includes both the raw inputs and any intermediate artifacts the analysis consumed. One needs the *code*, along with its version and its dependencies, in enough detail that another investigator knows exactly which operations were run. And one needs the *runtime environment*: the interpreters, compilers, operating-system facilities, and libraries that the code invoked. All three are necessary, and none is sufficient on its own.

Of the three, the runtime environment has historically been the most difficult to preserve. Whereas data can be deposited in an archive and code pushed to a public repository, runtimes decay: libraries go out of support and operating-system versions cease to boot, and the hardware they assume changes underneath them. In computational biology, this problem of runtime rot has been documented empirically. Early surveys of web-based bioinformatics tools noted that significant fractions of URLs in the literature became unreachable within a few years.^7^^,^^8^ A decade-scale follow-up found that roughly a quarter of nearly two and a half thousand bioinformatics web services studied were no longer available, with server-side tools particularly affected.^9^ The same pattern holds for downloadable tools: when Mangul et al.^10^ attempted to install 98 published omics tools in a fresh environment following each tool’s own documentation, 28% could not be installed at all, and nearly half failed the stricter “easy-to-install” test. In many of these cases, the source code was never the bottleneck: it was the runtime environment that could not be kept alive.

The community response has been to bring the runtime environment under the same version-controlled, archivable discipline applied to code. Container technologies such as Docker and Apptainer (formerly known as Singularity) package the full runtime environment together with the code, producing an artifact that another investigator can download and execute years later.^11^^,^^12^ Continuous-integration systems and package managers test software in specific environments and automate installation. Package-management ecosystems such as Anaconda and renv implement reproducible environments that can be specified declaratively and rebuilt on demand.^13^ Workflow managers such as Snakemake and Nextflow tie data, code, and a containerized runtime together into a single executable description of an analysis.^14^^,^^15^ These tools did not solve the runtime problem, but they made the runtime environment a citable, archivable artifact.

## LLMs as runtimes

A C program is a human-readable text file of instructions; the compiler turns those into machine-readable instructions for a processor. An LLM prompt is also a text file of instructions; a language model is the thing that runs it. Seen this way, the same questions one would ask about any piece of software (what version of the interpreter, what inputs does it expect, what happens when it is updated) apply to prompts and other LLM instructions, including skills.

Each prompt, skill, or API-call instruction handed to an LLM-based system during an analysis prescribes behavior, and the system produces behavior in response. In contemporary agents, a harness processes that instruction and combines it with system instructions, conversation history, tools, and other context before invoking the model. This contrasts with earlier chat interfaces, which typically passed the user’s prompt and conversation history to the model with less additional context and processing. The model, together with its harness, is the runtime environment for those instructions. This framing organizes familiar questions about versioning, inputs, outputs, and updates in a way that scientists already have experience reasoning about for compilers and interpreters. There is, however, no standardized way for investigators to ensure or communicate reproducibility of LLM-based analyses.

Data analysis programs can be designed to be deterministic. LLM inference, particularly through hosted services, is not guaranteed to be deterministic. Even with settings chosen to reduce randomness (a sampling temperature of zero and the same random seed each time), outputs can vary across invocations because of small numerical effects in the inference stack: the order of operations on the hardware, the batching of multiple requests, the specific sampling implementation.^16^^,^^17^ These variations are small in each step but compound across long workflows, so that a prompt executed twice against the same model may not produce identical output.

A second break with pre-LLM workflows is the provenance of the runtime environment itself. Compilers and interpreters used to translate analysis code into machine-executable code are open and archivable: another investigator can, in principle, reconstruct the exact binary. The weights of a closed-weight language model are not. Commercial LLM vendors do not typically guarantee that a model available today will be unchanged a day, let alone a year, from now, and frontier models change in ways that are opaque to users but that influence output. A skill, prompt, or API call written against a specific closed-weight model is, in effect, code whose runtime environment may be changed or deprecated at any time after use.

A third consideration is the harness. Model outputs depend heavily on the harness used to access data (e.g., through Model Context Protocol (MCP) servers), tools available to the model, and the way context is assembled and managed. Context windows range from thousands to millions of tokens but fill quickly with data, codebases, literature, and prior exchanges. As the context grows, a harness may summarize or “compact” earlier material, truncate it, or selectively retrieve information. These context-management procedures are governed by harness heuristics that can change frequently, as Claude Code’s near-daily releases illustrate.^18^ Harness internals are typically not exposed to the user and often cannot be pinned to a version, so the same prompt and model can produce different output simply because context management has shifted underneath the user. This opacity is itself a limit on reproducibility, and one that users of hosted systems are rarely in a position to remove.

## Roles of LLMs in analysis

LLMs play several roles in scientific data analysis, and the data-path distinction (Figure 1) applies differently in each.

Whether an LLM operates on or off the data path depends on how the path is defined relative to the claims of the paper. Here, we define the data path as the sequence of operations that transforms declared inputs into the outputs evaluated in the paper. Hypothesis generation could therefore usually be considered to be off-path. It becomes on-path when the LLM-generated output is itself evaluated, presented as a methodological result, or used directly to generate a reported result. The same activity may consequently be off-path in a biological application paper and on-path in a paper evaluating the agent itself. Where and how to draw these lines is a thorny epistemological topic that will require attention in the near future.

In the following sections we group the roles by where the LLM sits.

## Off the data path

### Code generation and modification

A scientist prompts a language model to write, refactor, or debug software that is then incorporated into the analysis. The model sits off the data path (Figure 1A). The LLM-specific runtime dependency is removed. What remains is a durable artifact, usually code executed by a conventional runtime, subject to established practices for preserving data, code, and runtime. Much biological software is written by domain scientists without formal training in software engineering, and the community has been slow to adopt practices such as systematic testing, code review, and exploration of alternative implementations^19^; LLM coding assistants lower the cost of these practices, and reproducibility and robustness may improve when the practices are actually applied. There are also critical failure modes in what Karpathy termed *vibe coding*^20^: accepting LLM-generated code without close review and iterating by prompting rather than by reading. Vibe coding can produce code at a scale investigators could not otherwise reach, and the risk is that it is not sufficiently evaluated for correctness or even understood by the creator. LLMs do not replace the need for scientists to know how to write and understand code.

## On the data path

### Orchestration

A language model reads a high-level description of a workflow and interacts with a harness that invokes the tools, scripts, and services that perform the work, deciding each step at run time. The LLM is on the data path; without it, the analysis does not run. If the agent’s orchestration logic is recorded, it can be committed as a pipeline (e.g., Snakemake^14^ or Nextflow^15^), which removes the LLM from the data path.

### Data munging

Cleaning typos, enforcing a schema, normalizing units, and reconciling inconsistent records are natural targets for language models because the work is tedious, heterogeneous, and often just past the reach of a regular expression. (A sample-sheet column with male, M, male, and male (from freezer 3) is easy for a model but takes dozens of regex branches.) The transformation is the model’s output, so the LLM sits on the data path (Figure 1B), where it may apply transformations the user did not intend. An alternative off-path approach would be for the LLM to identify formatting issues in the data and design programmatic transforms that the user can inspect before they are applied to the data.

### Direct analysis

Language models can perform tasks that previously required specialized software: transcribing audio, extracting bounding boxes, classifying documents, reading plots, and summarizing free-text records. The LLM may operate on the data directly, or invoke a specialized non-LLM system (e.g., an image-segmentation network) that does. If the LLM is required at run time to select, configure, or interpret that operation, the step fails the rerun-without-LLM test. The LLM remains on the data path unless its role can be reduced to fixed, deterministic code. For example, Castro et al.^21^ used large language models to extract species-distribution data from unstructured news reports and papers. The models classified documents, identified the regions in which species were recorded, and returned structured coordinates.

### Skills as a packaging of instructions

Across these roles, instructions to language models are increasingly packaged in structured, reusable formats. Anthropic’s Agent Skills^22^ are one example: a skill is a directory containing an SKILL.md file (a short description plus a body of instructions) alongside optional markdown or executable scripts. The agent loads only the description into context, and loads the full body and supporting files on demand, a mechanism called *progressive disclosure*. Markdown is often more accessible to scientists than a software package or workflow language, and a scientist with modest technical background can write a useful skill far more readily than a Python package or a Snakemake workflow. Making skills shareable does not solve reproducibility challenges intrinsic to the LLM itself.

Skills can be written to place the LLM on or off the data path. For a common bioinformatics task (filter a FASTA file to sequences of at least N bases), one skill body might say “*Emit the header and sequence to the output for every record at least N bases long*,” while another says “*Write a Python script that does this*, *then run it*; *do not read the FASTA yourself*.” The first puts the model on the data path: reproducing the output requires the same model. The second puts the model off it: the committed artifact is a script executed by a conventional runtime that can be versioned and archived. The same task; different placement.

## Provenance, determinism, and interpretability when the LLM is on the data path

When the LLM remains on the data path, the workflow acquires additional reproducibility constraints because the model and harness may not be archivable or stable. Three properties remain at least partly under the authors’ control: how well the behavior of the LLM at the time of analysis is recorded (*provenance*), how stable that behavior is across repeated runs (*determinism*), and how accessible the internal steps are to inspection (*interpretability*).

Provenance is a structured record of how a result was produced.^23^^,^^24^ For an LLM step on the data path, a useful minimum record includes the model name and version, harness information where the system exposes it (it is often obfuscated from the user), the prompt or skill used (with a version where applicable), any sampling parameters (temperature, top-p, seed), and any other inputs that influence behavior. Capturing this information is cheap and easy to automate. It does not solve reproducibility on its own (a closed-weight model may be retired regardless of how well its version was recorded), but it is what makes any later forensic audit possible.

Determinism is a separate question, and it is useful to distinguish it from a related property. *Within-runtime determinism* is the extent to which repeated invocations of the same model with the same inputs produce the same output; it can be characterized by running the step many times and comparing outputs. Even with ostensibly deterministic settings, models often show non-trivial variation.^16^^,^^17^ *Cross-model agreement* is a distinct property: the extent to which the same prompt or skill produces equivalent outputs against different models, ideally from different vendors. This is not determinism, since different models are different systems, but it is harder to achieve and provides stronger insurance against a specific runtime environment being retired. Neither property admits a pass-fail threshold: some analyses tolerate substantial stochasticity because downstream logic absorbs the variation; others require bit-for-bit determinism and are poorly served by any current LLM-on-data-path approach. The useful recommendation is simple: if the LLM is on the data path, record its version and evaluate both determinism and cross-model agreement.

Interpretability is the extent to which the internal steps a method took to reach its output can be inspected and understood. It sits at a different angle from the first two properties: provenance asks what was recorded about an LLM step, and determinism asks whether that step repeats, while interpretability asks what happened inside it. Steps a model takes in context without issuing a tool call remain opaque regardless of whether its weights are open or closed. Whether that opacity matters depends on the role: a method does not have to be interpretable to be useful.^25^ For tasks whose output is itself a prediction (classifying an image, extracting a bounding box), opacity is tolerable if the method is well-characterized on held-out data. For tasks where the output is a claim about how or why something happens, opacity is more serious, because the claim is precisely the thing another investigator would otherwise audit. Either way, when internal steps are opaque the burden of verification shifts to external assessment of the output (addressed in the section on verification).

One mitigation strategy is to have the model keep a lab notebook: record structured notes, tool calls, intermediate outputs, and decision rationales alongside its final outputs. Agent frameworks can often generate such execution records automatically, and they are cheap to archive. The caveat is that a model’s written explanation is not guaranteed to match the computation that produced its output; chain-of-thought explanations can systematically misrepresent the factors that drove a prediction.^26^ Rationales are therefore the model’s claims about its process, whereas tool calls and intermediate artifacts document externally observable steps. Verification should still test the output.

## Verification of LLM results

A separate question from whether an LLM step is reproducible is whether its output is correct. LLMs can produce outputs that are wrong in subtle or substantive ways: hallucinated text,^27^ incorrect classifications, faulty extractions, or unstable judgments. An analysis that includes an LLM step therefore needs to verify what the LLM produced and document how, and the data-path distinction reshapes verification sharply.

### Off the data path

When the LLM is used to write code, the output can be verified directly with standard software-engineering approaches: unit tests, integration tests, property-based tests, static analysis, regression tests, and code review. A unit test that exercises code against known inputs and expected outputs is the same artifact whether the code was written by a human, an LLM, or both. The risk in LLM-assisted code development comes from failing to actually perform these verifications (the vibe-coding failure mode noted earlier), not from the absence of mature tools to do them.

### On the data path

When the LLM is itself a node on the data path, verification is harder: the output of the LLM step is exactly what would require ground truth to evaluate, yet ground truth is rarely available at the scale needed. The practical response is a portfolio of partial checks chosen to fit the task. *Held-out benchmark data* provide a quantitative estimate of performance where labeled examples exist, the approach underlying standard LLM benchmarks^28^; for frontier closed-weight models, public benchmarks may already be in the training data,^29^ so private or contamination-resistant fixtures give a more honest read. *Test fixtures with known ground truth* are a smaller, committed analogue: a hand-curated panel that exercises the specific behaviors the analysis depends on and can be rerun as the model or prompts change. *Manual spot-checks* of a random sample, with sample size chosen to give a target error bound, are inexpensive when the task is easy for a human to judge but tedious to do at scale. *Agreement statistics* such as Cohen’s kappa quantify concordance between the LLM and human annotators on tasks where humans themselves disagree, while classification metrics such as precision, recall, and F1 apply when one annotation set is treated as the reference. *Cross-method agreement* compares the LLM step with a non-LLM algorithm or classical statistical method; when such independent methods are available, it is usually preferable to place them, rather than the LLM, on the data path. *LLM-as-judge*^30^ uses a second, ideally different-vendor model to evaluate the first against a rubric; it scales where manual checking cannot but imports its own failure modes. *Sensitivity tests* perturb inputs (reword a prompt, rotate an image, and replace a synonym) to check that small input changes produce proportionate output changes; this overlaps with evaluation of determinism.

### Formal verification

Some applications require stronger assurance than test suites in software development provide: even complete structural code coverage can miss faults triggered by untested inputs.^31^ In mathematics, this stronger assurance can take the form of a formal proof that establishes a quantified claim for every input rather than only tested examples. LLMs are increasingly used to derive such proofs, and proof-checking frameworks such as Lean^32^ can check their validity regardless of whether a proof was written by a human or a model. A proof accepted by the checker is valid relative to the formal statement and its assumptions, although this does not establish that the formalization accurately captures the original question. Here, trust and reproducibility rest on the verification tool, such as Lean, which is itself deterministic and archivable, rather than on the model that generated the proof.

No single check is sufficient for most on-path uses, and applications will usually combine several. The choice of verification methods is itself a design decision; the methods used and what they found should be reported alongside the analysis so another investigator can judge what was verified and what was not.

## Recommendations

We draw six recommendations from the preceding sections.

### Place the LLM off the data path where possible

Off-path placement keeps the workflow within the familiar framework of conventional computational reproducibility: archivable source, snapshottable runtime, and re-executability (see the section on reproducibility). On-path placement adds the LLM as a runtime dependency, with weaker reproducibility properties. On-path use is not intrinsically problematic. It may be worthwhile when the relevant capability cannot readily be reduced to a fixed, inspectable pipeline for the application at hand, as with classifying images, extracting bounding boxes, reconciling heterogeneous records, or interpreting a plot; in such cases the additional reproducibility burden may be worth paying. But where the same task could equally well be accomplished by code, including code generated by an LLM, leave the LLM off the path. Put simply, do not use an LLM to produce results you could trivially obtain from LLM-written code. The rerun-without-LLM test (introduced in the introduction) is a sharp check: if the published analysis can be re-executed end-to-end with no model calls, the LLM is off the path, and reproducibility no longer depends on access to the LLM. In that case, recording the details of LLM use (which model, which prompts) is not required for reproducibility, though it may still be warranted by publication policy or documentation norms.

### Preserve LLM-related artifacts in a versioned, archived repository

When the LLM is off the data path, the additional preservation requirement falls primarily on the durable artifacts that generate the published result. This is typically the code produced with the assistance of an LLM. In this case, prompts, skills, and other records of interaction with the model may still be useful for provenance or documentation, but they are not usually required to rerun the analysis. When the LLM is on the data path, the preservation burden is stronger. The instructions given to the model at run time, such as prompts embedded in API calls, skills loaded from a repository, schemas, and similar inputs, as well as the artifacts the model produces, such as invocation records, intermediate outputs, and other inspectable metadata that affect execution, should be versioned and archived where possible.

### Verify LLM results, and document how

When the LLM is off the data path, apply standard software-engineering verification to the code it produces: unit tests, integration tests, code review, and regression tests. When the LLM is on the data path, use a portfolio of task-appropriate checks, such as held-out benchmarks, test fixtures with known ground truth, manual spot-checks on a random sample, inter-rater or cross-method agreement, LLM-*as*-judge, and sensitivity or adversarial tests. Report which checks were performed, what they found, and where remaining uncertainty lies (see the section on verification).

### Consider open-weight models

A model whose weights are published^33^^,^^34^^,^^35^^,^^36^ can, in principle, be retrieved and rerun by others later, giving the runtime a snapshottable character more like that of conventional software environments and narrowing the shelf-life gap that closed-weight models create. Open weights alone are not sufficient for an exact rerun, which may also depend on the tokenizer and configuration, inference software, hardware, and sampling parameters. Community efforts such as Marin^37^ push this further, opening the training data and procedures alongside the weights. Choosing an open-weight model does not necessarily require expensive local hardware; multiple providers host open models via an API. Closed-weight models, meanwhile, often perform better or offer features open models lack, so we do not make a blanket recommendation against closed-weight models. This landscape may look entirely different within several years as capabilities from today’s large frontier models are distilled into models that can run on smaller machines, or as computing resources capable of supporting cutting-edge or good-enough AI become cheaper and more equitably accessible.

### Record the model and its version at the time of analysis

For each inspectable LLM invocation on the data path, the minimum useful provenance record includes the model name and version, the harness and its version where the system exposes them, the sampling parameters (temperature, top-p, and seed where applicable), and the timestamp of the call. This information is cheap to capture and easy to automate. It does not solve reproducibility on its own. A deprecated closed-weight model is gone regardless of how carefully its version was recorded. Even so, recording this information is critical to any later forensic analysis. Where the model emits intermediate notes, reasoning traces, or step-by-step justifications as part of its work, archiving those alongside the results extends the provenance record at little additional cost, subject to the faithfulness caveat discussed earlier.

### Measure determinism within a model, and agreement across models

Both are empirical properties of an LLM step on the data path that are worth quantifying (discussed in the section on provenance and determinism): repeated invocations against the same model give within-runtime determinism, while comparisons against different models give cross-model agreement. Neither measurement is costless, but both become more valuable the more load-bearing the LLM step is. Where determinism is low and cannot be improved, downstream logic should be designed to tolerate the variation; where determinism matters and the task does not permit it, an on-path LLM step may not be the right tool, and the analysis may need to be restructured.

## Discussion

**Figure 2:**
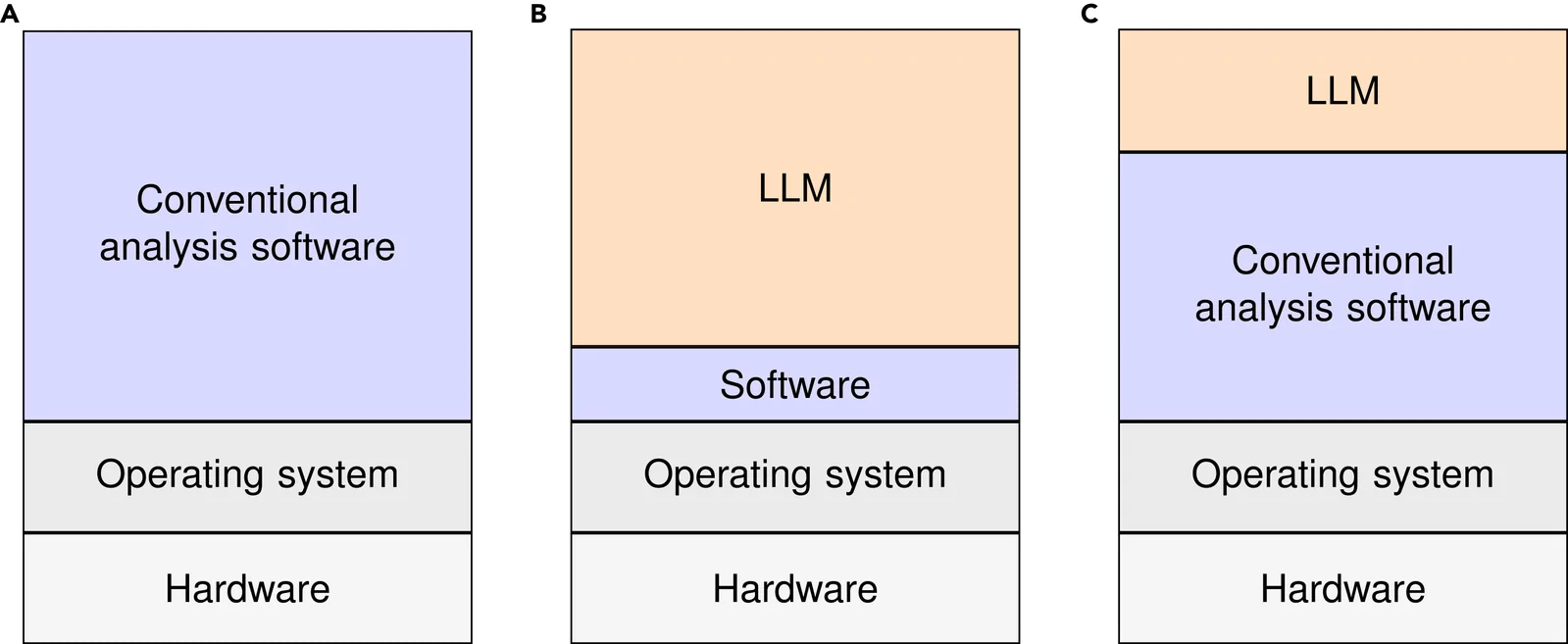
Three analysis stacks with different allocations of work between conventional software and an LLM. Hardware and operating system are held constant; what varies is how much of the analysis is implemented as code versus delegated to an LLM at run time. The LLM layer corresponds to on-path computation, while the software layer may include code authored off-path (A) Contains no LLM component. (B) Delegates most of the analysis to an on-path LLM. (C) Retains a substantial software layer with a bounded LLM component above it.

The tradeoffs that bear on LLM workflow placement decisions can change over the life cycle of a single analysis. During early exploration, when the scope is still forming and rapid iteration matters, leaning heavily on an LLM may be the right approach. This could favor a stack that looks like stack B in Figure 2. A scientist can reach a rough answer and try different angles of a dataset faster than with a stack that requires every step to be coded first. As findings stabilize and the work moves toward publication, the same analysis can be tightened: on-path invocations useful during exploration can be moved off-path and re-implemented as committed code, shifting the stack toward stack C or even A. The same progression occurs in traditional bioinformatic workflows, and planning for it is part of the rigorous design of any published computational analysis.

This new approach of using on-path LLMs to rapidly prototype analyses and then committing a clean, off-path pipeline at publication carries old statistical hazards. The published artifact can pass every reproducibility check while concealing problems with how it was reached. An investigator who favors a particular result can reprompt until that result appears, or simply ask the model to design an analysis that produces it; the committed code then reruns perfectly even though the result is a cherry-picked outlier. This is a fishing expedition, the practice known as p-hacking, with the search and design now automated and largely hidden.^38^ A subtler problem can arise even without any deliberate search: an agent asked to analyze a dataset makes a long cascade of reasonable but data-dependent choices (e.g., which records to drop, which transformation to apply, which test to run) that are never enumerated and that different data would have changed. That is the *garden of forking paths*.^39^ An opaque agent can traverse it at a scale, and with a lack of visibility, that a person working by hand cannot match. These problems are not created by LLMs; they are long-standing, but LLMs can make them easier to fall into and harder to detect.

LLMs also have the potential to address and reduce these problems. The same ease that makes fishing cheaper also makes defenses against it cheaper: an agent can rerun an analysis across many reasonable specifications, apply several independent methods to the same question, or repeat a step across different models. Used this way, LLMs help surface the very fragility that fishing and forking paths conceal. Other familiar mitigations still apply, including preregistration, multiverse-style reporting, or at a minimum documenting the range of approaches explored and how their results differed from the published one.

The recommendations aforementioned are author-facing, and whether practices like them take hold depends in large part on the standards that journals, preprint servers, and funders come to set for what an LLM-assisted manuscript reports. We do not propose such a standard here. The reporting frameworks that have proven durable in other domains, such as CONSORT for randomized trials,^40^ MIAME for microarray experiments,^41^ and ARRIVE for animal research,^42^ emerged from sustained, community-based development, and turned informal good practice into structured requirements only once the underlying science had matured enough to support them. Community-led efforts in this direction are already underway.^43^

Some of the raw materials that such efforts, directed at LLM-assisted research, might draw on already exist. Structured disclosure of LLM use is beginning to supplement the free-text “AI use” paragraph common at the end of many manuscripts.^44^ Declared software environments, such as a conda specification or an equivalent manifest pinning library versions, are already routine lightweight reproducibility artifacts in computational fields and carry over directly. Reproducibility badges adopted by several journals^45^ recognize stronger forms of reproducibility without penalizing authors who cannot yet reach them. New scientific workbenches are beginning to address these challenges by recording code, environments, and auditable histories alongside their outputs. Claude Science, for example, pairs each generated figure with the code and environment that produced it, a plain-language description of how it was made, and the full message history.^46^ Tools of this kind close much of the provenance gap described previously, though the model they invoke remains closed-weight and vendor-controlled: they improve the record of an analysis without extending the shelf life of its runtime. We expect such tools to become increasingly common as these systems mature. Where an on-path LLM step is load-bearing, the determinism measurements described earlier—repeated invocations on the same input and agreement across models—are cheap enough to report routinely. Shared, contamination-resistant benchmark datasets and ground-truth fixtures for common on-path tasks, still rare in many biological domains, are another resource such efforts could build, giving authors a common ground truth against which to report the accuracy of an LLM step.

### Conclusion

The framework here turns on a single design choice: whether an LLM sits on the data path of the published analysis or off it. Off-path placement leaves reproducibility resting on archived data, code, and a conventional runtime; on-path placement adds the LLM’s runtime properties, which are weaker. When to record provenance, how to verify outputs, how to measure determinism: the answers fall out of this placement.

The rerun-without-LLM test is the operational form of the distinction between on-path and off-path LLM use. An analyst who can re-execute the published analysis end-to-end with no model calls has an off-path workflow, where reproducibility depends on the archived data, code, and conventional runtime rather than on continued access to the LLM; an analyst who cannot has taken on additional obligations, because the live LLM is part of the analysis. The same formal properties still matter; they just require different forms of record-keeping and verification.

The useful question to ask of a data analysis workflow is therefore not how it was produced but what properties it has. A pipeline hand-written in C and a pipeline assembled by prompting a language model can each be correct or incorrect, shelf-stable or ephemeral, deterministic or not, interpretable or opaque, well-provenanced or not, and those properties are what determine whether the work can be trusted and built upon. Reproducibility, determinism, provenance, and interpretability are no less relevant for a prompt, skill, or API call executed by a language model than they are for C code compiled for a particular CPU. Computer science and software engineering have been working on these properties for decades; their frameworks do not need to be replaced, only extended. The tools are different but the principles remain. Establishing these practices during the early adoption of LLMs in scientific analysis, while the tools remain in flux and the stakes of individual analyses remain manageable, is how the reproducibility work of the past several decades carries forward into the next.

## Acknowledgments

We thank Roy Lederman for helpful comments on an earlier draft of this manuscript.

## Author contributions

C.W.D.: conceptualization, writing – original draft, writing – review and editing. D.T.S.: writing – original draft, writing – review and editing. J.M.M.: writing – original draft, writing – review and editing.

## Declaration of interests

The authors declare no competing interests.

## Declaration of generative AI and AI-assisted technologies in the writing process

During the preparation of this work, the authors used Claude (Anthropic; models Opus 4.7, 4.8, and 5) and ChatGPT (OpenAI; models GPT 5.3, 5.4, and 5.6) to assist with drafting and copy-editing the manuscript text, checking and formatting references, and writing the TikZ code that generates the figures. The authors reviewed and edited the output as needed and take full responsibility for the content of the published article.
